# Supplementary material for: Measurement of Motivation States for Physical Activity and Sedentary Behavior: Development and Validation of the CRAVE Scale
Source: Front Psychol. 2021 Mar 25;12:568286. doi: 10.3389/fpsyg.2021.568286 (PMC8027339; doi:10.3389/fpsyg.2021.568286)
Supplement: Supplementary file 1 [file Table_1.DOCX]

| Supplemental Table 1. Descriptive statistics (means, SD) and inter-item correlations for CRAVE items assessed “right now” (Study 2 data) | | | | | | | | | | | | | | | | | | |
| --- | --- | --- | --- | --- | --- | --- | --- | --- | --- | --- | --- | --- | --- | --- | --- | --- | --- | --- |
|  |  | Mean | SD | 1 | 2 | 3 | 4 | 5 | 6 | 7 | 8 | 9 | 10 | 11 | 12 | 13 | 14 | 15 |
| 1 | move my body | 5.32 | 3.02 | 1 |  |  |  |  |  |  |  |  |  |  |  |  |  |  |
| 2 | be physically active | 5.56 | 3.15 | .81** | 1 |  |  |  |  |  |  |  |  |  |  |  |  |  |
| 3 | do nothing active | 4.23 | 3.28 | -.51** | -.57** | 1 |  |  |  |  |  |  |  |  |  |  |  |  |
| 4 | just sit down | 5.11 | 3.23 | -.58** | -.55** | .65** | 1 |  |  |  |  |  |  |  |  |  |  |  |
| 5 | burn some calories | 5.68 | 3.35 | .54** | .59** | -.37** | -.39** | 1 |  |  |  |  |  |  |  |  |  |  |
| 6 | "veg out" (vegetate) | 3.06 | 3.06 | -.01 | .02 | .11* | .13** | .01 | 1 |  |  |  |  |  |  |  |  |  |
| 7 | expend some energy | 5.33 | 2.88 | .73** | .73** | -.50** | -.54** | .62** | 0.09 | 1 |  |  |  |  |  |  |  |  |
| 8 | be still | 4.34 | 3.21 | -.58** | -.54** | .65** | .69** | -.40** | .23** | -.49** | 1 |  |  |  |  |  |  |  |
| 9 | be a couch potato | 3.69 | 3.33 | -.51** | -.54** | .65** | .64** | -.39** | .22** | -.47** | .69** | 1 |  |  |  |  |  |  |
| 10 | walk about | 4.96 | 2.81 | .61** | .54** | -.36** | -.36** | .39** | .10* | .57** | -.36** | -.38** | 1 |  |  |  |  |  |
| 11 | exert my muscles | 5.35 | 2.93 | .70** | .74** | -.50** | -.54** | .54** | .04 | .73** | -.47** | -.48** | .54** | 1 |  |  |  |  |
| 12 | be motionless | 3.65 | 3.19 | -.57** | -.56** | .62** | .66** | -.44** | .28** | 0.52** | .79** | .72** | -.37** | -.50** | 1 |  |  |  |
| 13 | lay down | 5.92 | 3.38 | -.47** | -.51** | .57** | .60** | -.31** | .11* | -.41** | .60** | .63** | -.35** | -.43** | .58** | 1 |  |  |
| 14 | rest my body | 6.05 | 3.31 | -.47** | -.49** | .56** | .65** | -.33** | .13** | -.43** | .62** | .60** | -.29** | -.44** | .60** | .84** | 1 |  |
| 15 | move around | 5.54 | 2.98 | .72** | .70** | -.48** | -.52** | .56** | .03 | .69** | -.54** | -.46** | .66** | .68** | -.53** | -.44** | -.44** | 1 |
| * *p*< .05, ** *p*< .01 | | | | | | | | | | | | | | | | | | |
