# Supplementary material for: Measurement of Motivation States for Physical Activity and Sedentary Behavior: Development and Validation of the CRAVE Scale
Source: Front Psychol. 2021 Mar 25;12:568286. doi: 10.3389/fpsyg.2021.568286 (PMC8027339; doi:10.3389/fpsyg.2021.568286)
Supplement: Supplementary file 2 [file Table_2.DOCX]

| Supplemental Table 2. Descriptive statistics (means, SD) and inter-item correlations for CRAVE items assessed “in the past week” (Study 1 data) | | | | | | | | | | | | | | | | | | |
| --- | --- | --- | --- | --- | --- | --- | --- | --- | --- | --- | --- | --- | --- | --- | --- | --- | --- | --- |
|  |  | Mean | SD | 1 | 2 | 3 | 4 | 5 | 6 | 7 | 8 | 9 | 10 | 11 | 12 | 13 | 14 | 15 |
| 1 | move my body | 7.26 | 2.08 | 1 |  |  |  |  |  |  |  |  |  |  |  |  |  |  |
| 2 | be physically active | 7.53 | 2.13 | .68** | 1 |  |  |  |  |  |  |  |  |  |  |  |  |  |
| 3 | do nothing active | 3.04 | 2.6 | -.30** | -.41** | 1 |  |  |  |  |  |  |  |  |  |  |  |  |
| 4 | just sit down | 4.33 | 2.61 | -.22** | -.28** | .61** | 1 |  |  |  |  |  |  |  |  |  |  |  |
| 5 | burn some calories | 7.16 | 2.42 | .40** | .55** | -.23** | -.15** | 1 |  |  |  |  |  |  |  |  |  |  |
| 6 | "veg out" (vegetate) | 3.56 | 2.87 | .07 | .06 | .11* | .09* | .20** | 1 |  |  |  |  |  |  |  |  |  |
| 7 | expend some energy | 6.84 | 2 | .53** | .57** | -.33** | -.29** | .48** | .23** | 1 |  |  |  |  |  |  |  |  |
| 8 | be still | 3.34 | 2.62 | -.25** | -.24** | .52** | .54** | -.21** | .16** | -.23** | 1 |  |  |  |  |  |  |  |
| 9 | be a couch potato | 3.01 | 2.8 | -.37** | -.37** | .54** | .49** | -.24** | .14** | -.29** | .50** | 1 |  |  |  |  |  |  |
| 10 | walk about | 5.95 | 2.42 | .27** | .21** | -.14** | -.06 | .30** | .13** | .33** | -.02 | -.14** | 1 |  |  |  |  |  |
| 11 | exert my muscles | 6.89 | 2.35 | .48** | .64** | -.35** | -.32** | .48** | .16** | .56** | -.18** | -.25** | .24** | 1 |  |  |  |  |
| 12 | be motionless | 2.31 | 2.39 | -.37** | -.38** | .55** | .50** | -.29** | .16** | -.32** | .62** | .59** | -.14** | -.27** | 1 |  |  |  |
| 13 | lay down | 5.42 | 2.8 | -.12** | -.11* | .34** | .45** | -.10* | .06 | -.15** | .34** | .44** | .01 | -.16** | .33** | 1 |  |  |
| 14 | rest my body | 5.95 | 2.63 | -.14** | -.12* | .32** | .39** | -.06 | .04 | -.09** | .32** | .37** | -.06 | -.11* | .31** | .75** | 1 |  |
| 15 | move around | 7 | 2.15 | .56** | .53** | -.37** | -.28** | .41** | .10* | .57** | -.22** | -.32** | .44** | .48** | -.34** | -.08 | -.07 | 1 |
| * *p* < .05, ** *p* < .01, *** *p* < .001 | | | | | | | | | | | | | | | | | | |
