# Supplementary material for: Measurement of Motivation States for Physical Activity and Sedentary Behavior: Development and Validation of the CRAVE Scale
Source: Front Psychol. 2021 Mar 25;12:568286. doi: 10.3389/fpsyg.2021.568286 (PMC8027339; doi:10.3389/fpsyg.2021.568286)
Supplement: Supplementary Data Sheet 1 — The scale development. [file Data_Sheet_1.docx]

**Supplement 1: Scale Development.**

The overarching goal was to create a brief and non-burdensome instrument (< 20 items), grounded in theory, with adequate reliability and construct validity (1-6). In designing the instrument, two general considerations were: 1) to align with theoretical models of desire to move/rest (7), and 2) to keep the scale consistent with other validated scales of desire/craving (8, 9). Items for each part of the questionnaire were generated by three researchers (MSK, TG and RS) who have research experience in the areas of physical activity, kinesiology, and psychology of addictive behaviors.

The first task was to create an initial item pool with items selected based on criteria from Clark and Watson (10, 11). The intention was to be over-inclusive and even generate some items that may not factor well with the core idea of wants for muscular movement. Items were selected in consideration of the WANT model, which was being developedconcurrently (7), and in regards to their definition of motivation states for physical activity and rest. We aimed to consider 4 essential properties of desires/cravings from the WANT model (7) in this scale development: 1) desires for movement and rest reside on different, orthogonal dimensions in contrast to opposite sides of the same pole, and thus may operate asymmetrically, 2) desires for movement and rest might be described as either “wants” or “desires”, 3) desires vary in intensity, and may be perceived to be completely absent (have a 0 point), and 4) desires/wants have state-like traits; they are in “the present moment”. However, not included were the concepts of hedonic dread or aversions (“diswants”) for activity and rest, which while specified in the model are less developed concepts(12). It was also assumed that a lack of desire to partake in actions articulated in the sample questions (i.e., scoring near 0) would capture aversion states to those same actions.

In line with the WANT model, we evaluated desires for muscular movement at a broad level of abstraction (10, 11) that would include exercise, physical activity, non-exercise activity thermogenesis (NEAT) and sport - as well as sedentary states except for sleep. Items were created to indicate desires for actions (i.e., wanting to move the body) and not for feelings, emotions, or sensations (e.g., wanting to feel burning or tension in the muscles, wanting to enjoy a workout), physical activity preferences or other dimensions of exercise-related wants or goals (e.g., to gain fitness, to get stronger, to become leaner, to socialize or compete). Three basic qualities or dimensions were strongly considered, each perceptible to the individual/ kinesthetic awareness (13), that might result in experiential intrinsic motivation for the activity itself (14): a) the physical body in motion, b) contracting the musculature, c) burning kilocalories. Also considered were existing items from non-validated scales (see general introduction) (15-20) and supporting literature (21-23). We purposely avoided the use of the words “exercise” and “sport” in the scale, terms used by other researchers (24, 25) as these can refer to complicated sets of behaviors related to participation in sport training, competition, being a bystander, coach, referee, etc. or may refer to other non-relevant activities, such as merely being associated with a group.

This effort resulted in 28 items (15 for move, 13 for rest): move my body, be physically active, burn some calories, expend some energy, exert my muscles, move around, walk about, be aerobically active, activate my body, get up and go, get up and move, get up and move around, get moving, release energy, work on getting stronger, do nothing active, just sit down, be still, be a couch potato, be motionless, lay down, rest my body, “veg out” (vegetate), relax my muscles, be lazy, just “crash”, go to sleep, and release tension in my body (20). A process of item trimming was then undertaken by authors MSK and TG. The items were also provided to two sets of graduate students in sports medicine and applied physiology who evaluated the items based on the following dimensions from Clark and Watson (10, 11): adheres to theory, measures the intended construct, simple-language, easy to understand, non-trendy, and not being overly similar to other constructs. Thirteen items were identified as problematic and eliminated. Of the 15 remaining items, seven items related to being physically active (e.g., want/desire to burn some calories; be physically active; exert my muscles, etc.) while eight items were sedentary behaviors (e.g., want/desire to just sit down, do nothing active, rest my body, etc.). These were then checked for face validity by four expert scholars (noted for being experts in the field of exercise psychology) from four different institutions who all endorsed the items (JB, JC and two in the acknowledgements section).

Items were paired with an 11-point Likert Scale ranging from 0 = not at all, to 10 = more than ever (8, 26). We choose an 11-point item response to be a balance between too few responses and too many. Some authors have suggested that a 100-point system is better (9) while others suggest 5 to 7 is ideal (10, 11). The response item “more than ever” was chosen because we wanted to make sure that responses were gauged from a personal perspective and not in consideration of a theoretical maximum, which may have no meaningful point of reference for the respondent (8, 9). As for the instructions, it was decided to ask respondents to “indicate how much you want or desire to perform the following activities”. There is some controversy over whether to focus on the word “want” or “desire” in these types of assessments, with some researchers indicating the use of the word “desire” over “want” to minimize the impact of “shoulds”, the conflation with goals and intentions, and because there is some ambiguity over the meaning of the word “want” (27). However, other researchers have essentially equated wants and desires, indicating a high degree of overlap between the two concepts (7, 28). An additional instruction was also added: “Do not think about how much you ‘should’ desire each activity. Think about how much you actually want/desire…”. The scale, in its final form, was checked by a third group of applied physiology graduate students, the same 4 expert scholars and a small community sample for clarity and ease of understanding, a process which indicated the scale was ready for further validation.

| **Items in the CRAVE scale** | | | | |
| --- | --- | --- | --- | --- |
|  | FINAL | Round 3 item trimming | Round 2 item trimming | Round 1 item trimming |
| **GRAND INITIAL TOTAL - 28 ITEMS** | Final CRAVE items (10 remaining) | SUBMISSION 1 explanation:  These items deleted after second EFA analysis (3 more trimmed)  SUBMISSION 2 explanation:  Eliminated because of low correlations < .3 (PW)  (29) | SUBMISSION 1 explanation:  These items deleted after first EFA analysis (2 more trimmed)  SUBMISSION 2 explanation:  Lots of non-significant correlations for Past Week part (not Right now) | These items disregarded and no data collected (13 trimmed) |
| MOVE (15 total items originally) | move my body, be physically active, expend some energy, exert my muscles, move around | burn some calories | walk about | be aerobically active, activate my body, get up and go, get up and move, get up and move around,  get moving, release energy,  work on getting stronger |
| REST  (13 total items originally) | do nothing active, just sit down  be still, be a couch potato, be motionless | lay down, rest my body | “veg out” (vegetate) | relax my muscles, be lazy,  just “crash”, go to sleep, release tension in my body |

1. Watson D, Clark LA, Tellegen A. Development and validation of brief measures of positive and negative affect: the PANAS scales. Journal of Personality and Social Psychology. 1988;54(6):1063-70.

2. Bohn MJ, Krahn DD, Staehler BA. Development and initial validation of a measure of drinking urges in abstinent alcoholics. Alcohol Clin Exp Res. 1995;19(3):600-6.

3. Sayette MA, Wilson SJ. The measurement of desires and craving. In: Hofmann W, Nordgren L, editors. The Psychology of Desire. New York, NY: Guilford Press; 2015.

4. Singleton EG, Tiffany ST, Henningfield JE. Alcohol Craving Questionnaire (ACQ-NOW): Background, Scoring, and Administration (Manual). Baltimore, MD: Intramural Research Program, National Institute on Drug Abuse; 2000.

5. Tiffany ST. A critique of contemporary urge and craving research: Methodological, psychometric and theoretical issues. Advances in Behaviour Research and Therapy. 1992;14(3):123-39.

6. Sinha R. The clinical neurobiology of drug craving. Current Opinion in Neurobiology. 2013;23(4):649-54.

7. Stults-Kolehmainen MA, Blacutt M, Bartholomew JB, Gilson TA, Ash GI, McKee PC, et al. Motivation States for Physical Activity and Sedentary Behavior: Desire, Urge, Wanting, and Craving. Frontiers in Psychology. 2020;11(3076).

8. Hommer RE, Seo D, Lacadie CM, Chaplin TM, Mayes LC, Sinha R, et al. Neural correlates of stress and favorite-food cue exposure in adolescents: a functional magnetic resonance imaging study. Hum Brain Mapp. 2013;34(10):2561-73.

9. Sayette MA, Shiffman S, Tiffany ST, Niaura RS, Martin CS, Shadel WG. The measurement of drug craving. Addiction. 2000;95 Suppl 2(Suppl 2):S189-210.

10. Clark LA, Watson D. Constructing validity: New developments in creating objective measuring instruments. Psychol Assess. 2019;31(12):1412-27.

11. Clark LA, Watson D. Constructing validity: Basic issues in objective scale development. Psychological Assessment. 1995;7(3):309-19.

12. Campese VD, Kim IT, Kurpas B, Branigan L, Draus C, LeDoux JE. Motivational factors underlying aversive Pavlovian-instrumental transfer. Learn Mem. 2020;27(11):477-82.

13. Grunbaum T. The body in action. Phenomenology and the Cognitive Sciences. 2008;7:243-61.

14. Stults-Kolehmainen MA, Gilson TA, Abolt CJ. Feelings of acceptance and intimacy among teammates predict motivation in intercollegiate sport. Journal of Sport Behavior. 2013;36(3).

15. Hutchinson JC, Sherman T, Davis L, Cawthon D, Reeder NB, Tenenbaum G. The influence of asynchronous motivational music on a supramaximal exercise bout. International Journal of Sport Psychology. 2011;42(2):135-48.

16. Pugh NE, Hadjistavropoulos HD. Is anxiety about health associated with desire to exercise, physical activity, and exercise dependence? Personality and Individual Differences. 2011;51(8):1059-62.

17. Paslakis G, Fauck V, Röder K, Rauh E, Rauh M, Erim Y. Virtual reality jogging as a novel exposure paradigm for the acute urge to be physically active in patients with eating disorders: Implications for treatment. Int J Eat Disord. 2017;50(11):1243-6.

18. Sartorius T, Heni M, Tschritter O, Preissl H, Hopp S, Fritsche A, et al. Leptin affects insulin action in astrocytes and impairs insulin-mediated physical activity. Cell Physiol Biochem. 2012;30(1):238-46.

19. Janata P, Peterson J, Ngan C, Keum B, Whiteside H, Ran S. Psychological and Musical Factors Underlying Engagement with Unfamiliar Music. Music Perception. 2018;36(2):175-200.

20. Casper RC, Voderholzer U, Naab S, Schlegl S. Increased urge for movement, physical and mental restlessness, fundamental symptoms of restricting anorexia nervosa? Brain Behav. 2020;10(3):e01556.

21. Mueller LM. The lived experience of intrinsic and extrinsic motivation in physical activity. 1982.

22. Rosa JP, de Souza AA, de Lima GH, Rodrigues DF, de Aquino Lemos V, da Silva Alves E, et al. Motivational and evolutionary aspects of a physical exercise training program: a longitudinal study. Front Psychol. 2015;6:648.

23. Rauch HGL, Schönbächler G, Noakes TD. Neural correlates of motor vigour and motor urgency during exercise. Sports Medicine. 2013;43(4):227-41.

24. Hofmann W, Baumeister RF, Forster G, Vohs KD. Everyday Temptations: An Experience Sampling Study of Desire, Conflict, and Self-Control. Journal of Personality and Social Psychology. 2012;102(6):1318-35.

25. Hofmann W, Vohs KD, Baumeister RF. What People Desire, Feel Conflicted About, and Try to Resist in Everyday Life. Psychological Science. 2012;23(6):582-8.

26. Jastreboff AM, Sinha R, Lacadie C, Small DM, Sherwin RS, Potenza MN. Neural Correlates of Stress- and Food Cue-Induced Food Craving in Obesity Association with insulin levels. Diabetes Care. 2013;36(2):394-402.

27. Williams DM, Bohlen LC. Motivation for exercise: Reflective desire versus hedonic dread. APA handbook of sport and exercise psychology, volume 2: Exercise psychology, Vol 2. APA handbooks in psychology series. Washington, DC, US: American Psychological Association; 2019. p. 363-85.

28. Kruglanski AW, Chernikova M, Rosenzweig E, Kopetz C. On motivational readiness. Psychol Rev. 2014;121(3):367-88.

29. Tabachnick BG, Fidell LS, Ullman JB. Using multivariate statistics: Pearson Boston, MA; 2007.
