# Supplementary material for: Measurement of Motivation States for Physical Activity and Sedentary Behavior: Development and Validation of the CRAVE Scale
Source: Front Psychol. 2021 Mar 25;12:568286. doi: 10.3389/fpsyg.2021.568286 (PMC8027339; doi:10.3389/fpsyg.2021.568286)
Supplement: Supplementary Data Sheet 2 — The actual scale. [file Data_Sheet_1.pdf]

CRAVE Scale

(Stults-Kolehmainen, M., Blacutt, M. et al.)

ID________ DATE________ TIME________

Indicate how much you **WANT** or **DESIRE** to perform the following activities by circling the number along each line between 0 (NOT AT ALL) and 10 (MORE THAN EVER).

Do NOT think about how much you "should" desire each activity.

Think about how you actually want/desire to behave in these ways **at this very moment (i.e., RIGHT NOW).**

At this very moment (right now) I want/desire to…

1) … move my body	NOT AT ALL	0-----1-----2-----3-----4-----5-----6-----7-----8-----9-----10	MORE THAN EVER
2) … be physically active	NOT AT ALL	0-----1-----2-----3-----4-----5-----6-----7-----8-----9-----10	MORE THAN EVER
3) … do nothing active	NOT AT ALL	0-----1-----2-----3-----4-----5-----6-----7-----8-----9-----10	MORE THAN EVER
4) … just sit down	NOT AT ALL	0-----1-----2-----3-----4-----5-----6-----7-----8-----9-----10	MORE THAN EVER
5) … burn some calories	NOT AT ALL	0-----1-----2-----3-----4-----5-----6-----7-----8-----9-----10	MORE THAN EVER
6) … expend some energy	NOT AT ALL	0-----1-----2-----3-----4-----5-----6-----7-----8-----9-----10	MORE THAN EVER
7) … be still	NOT AT ALL	0-----1-----2-----3-----4-----5-----6-----7-----8-----9-----10	MORE THAN EVER
8) … be a couch potato	NOT AT ALL	0-----1-----2-----3-----4-----5-----6-----7-----8-----9-----10	MORE THAN EVER
9) … exert my muscles	NOT AT ALL	0-----1-----2-----3-----4-----5-----6-----7-----8-----9-----10	MORE THAN EVER
10) … be motionless	NOT AT ALL	0-----1-----2-----3-----4-----5-----6-----7-----8-----9-----10	MORE THAN EVER
11) … lay down	NOT AT ALL	0-----1-----2-----3-----4-----5-----6-----7-----8-----9-----10	MORE THAN EVER
12) … rest my body	NOT AT ALL	0-----1-----2-----3-----4-----5-----6-----7-----8-----9-----10	MORE THAN EVER
13) … move around	NOT AT ALL	0-----1-----2-----3-----4-----5-----6-----7-----8-----9-----10	MORE THAN EVER

Scoring: The CRAVE has two subscales: Move and Rest. To calculate *move*, add the ratings from items 1, 2, 6, 9 and 13. To calculate *rest*, sum ratings from items 3, 4, 7, 8, 10. Unscored filler items are 5, 11 and 12.
